# Comparing the performance of the EQ-5D-3 L and the EQ-5D-5 L in an elderly Chinese population

**DOI:** 10.1186/s12955-020-01324-0

**Published:** 2020-04-09

**Authors:** Ruxu You, Jinyu Liu, Zhihao Yang, Chenwei Pan, Qinghua Ma, Nan Luo

**Affiliations:** 1 Department of Pharmacy, Union Hospital, Tongji Medical College, Huazhong University of Science and Technology, Wuhan, Hubei China; 2 Department of Pharmacy, Tongji Hospital, Tongji Medical College, Huazhong University of Science and Technology, Wuhan, Hubei China; 3 Health Services Mamagement Department, Guizhou Medical University, Guian, China; 4 School of Public Health, Medical College of Soochow University, Suzhou, China; 5 The 3rd People’s Hospital of Xiangcheng District, 568 Weizhong Road, Suzhou, 215134 China; 6 Saw Swee Hock School of Public Health, National University of Singapore, 16 Medical Drive, Block MD3, Singapore, 117597 Singapore

**Keywords:** EQ-5D-3 L, EQ-5D-5 L, Elderly population, Measurement properties, Quality of life

## Abstract

**Background:**

This study was conducted to compare the validity and discriminative power of both the EQ-5D-3 L and EQ-5D-5 L in an elderly Chinese population with multiple chronic and acute conditions.

**Methods:**

A total of 648 retired people from China (mean ± standard deviation: 73.3 ± 6.4 years; male: 55.7%) were recruited and randomized to complete the 3 L or 5 L questionnaire. The 3 L and 5 L were compared in terms of distribution properties, ceiling effects, informativity, validity and discriminatory performance. Convergent validity between the 3 L and 5 L was tested by spearman’s rank-order correlation. Discriminatory power was conducted by relative efficiency as assessed by the F statistics.

**Results:**

Most participants answered to “no problems” on both versions of EQ-5D. The 5 L trended towards a slightly lower ceiling compared with the 3 L. The Shannon index improved with the 5 L while the Shannon’s Evenness index tended to be similar. Convergent validity was confirmed by the moderate to strong correlation for both 3 L and 5 L. Relative efficiency suggested that 5 L had a higher absolute discriminatory power than the 3 L version in terms of the presence conditions, especially for osteoporosis and metabolic syndrome.

**Conclusions:**

Both the 3 L and 5 L are demonstrated to be valid based HRQoL instruments in Chinese elderly population. The 5 L system may be preferable to the 3 L, as it demonstrated superior performance with respect to lower ceiling effect and better discriminatory power. Further research is needed to examine the responsiveness of the two EQ-5D instruments in this population.

## Background

The EQ-5D is a generic preference-based measure of health related quality of life (HRQOL) that can be used for clinical and economic evaluation of health care as well as for the assessment of population health. It describes a respondent’s health status in five dimensions (mobility, self-care, usual activities, pain/discomfort and anxiety/depression) and produces a utility score anchored by 0 (death) and 1 (full health). The original version of the EQ-5D uses 3 severity levels (no problems, some problems, and extreme problems) to describe each dimension (EQ-5D-3 L). In 2009, EQ-5D-3 L was modified to have 5 response levels for each dimension (EQ-5D-5 L) in order to improve the instrument’s sensitivity and reduce ceiling effects [1]. Generally speaking, using measurement instruments with better measurement properties in comparative or evaluative studies is preferred. For example, more sensitive instruments are less likely to miss small but important between-group difference or with-group change. Regardless of the expected size of the difference or change, a smaller study sample would be needed in hypothesis-testing studies if a more sensitive instrument is used to measure the outcomes. Therefore, effort to improving the measurement properties of the EQ-5D instrument is practically meaningful.

Numerous studies have compared the psychometric performance of the EQ-5D-3 L (hereafter 3 L) and EQ-5D-5 L (hereafter 5 L). A recently published systematic review by Buchholz and colleagues found that compared to 3 L, 5 L has better ceiling effects and descriptive efficiency, as well as similar or better test-retest reliability [2]. It is not clear, however, whether 5 L is more sensitive than 3 L. Sensitivity can be assessed as sensitivity to change (or responsiveness) in longitudinal studies and sensitivity to difference (or discriminatory power) in cross-sectional studies. Buchholz and colleagues found that 5 L was more responsive than 3 L in 2 of 3 studies comparing the two EQ-5D instruments. Similarly, whether 5 L has higher discriminatory power compared to 3 L remains unclear. Although two recent studies in Greece demonstrated marginal to moderate better discriminatory power of 5 L compared to 3 L [3, 4]. Other studies found that 3 L performed better especially in discriminating between healthy and unhealthy populations [5–9]. One possible reason for the mixed results could be that the relative performance of 3 L and 5 L is population specific. Population characteristics such as age, race/culture, education level, language spoken, and health status may affect the performance of HRQOL instruments. For example, it is possible that 5 L is more sensitive than 3 L in patient populations but it is not more sensitive than 3 L in general elderly population because this population is generally healthy and tends to be inaccurate in describing their health due to lower literacy. Therefore, the relative performance of the two instruments in one population may not be generalizable to other populations and should be assessed for different populations individually.

Current research comparing the relative merits of 3 L and 5 L has two issues in study design. First, majority of the studies asked subjects to complete the 3 L and 5 L in one single survey, usually 5 L followed by 3 L. While such a within-subjects design is statistically efficient, it may suffer from the order effect, namely, responses to 3 L may be biased by the experience of responding to 5 L. Second, most of those comparative studies used a ‘crosswalk’ method to calculate the 5 L utility score [10, 11]. The ‘crosswalk’ method is an interim solution. The preferred method is to use a country-specific 5 L value set which was only made available in recent years for some countries. Studies have showed that utility scores derived from the ‘crosswalk’ method and value sets are not entirely equivalent and those may lead to different conclusions in cost-utility analysis [12, 13].

The purpose of the present study was to compare the performance of the 3 L and 5 L in health surveys of the elderly living in the community. The primary study aim was to assess the discriminatory power of the utility scores generated by the two EQ-5D instruments using a between-subjects design.

## Methods

This was a questionnaire-based, cross-sectional study of a general elderly population. The study was approved by the Ethics Committee, Medical College of Soochow University and followed the tenets of the Declaration of Helsinki (No20170720).

### Recruitment and data collection procedures

A consecutive sample of elderly residents who went to a community health center in a town near Suzhou city for routine health checkup was recruited from September to November 2017. Inclusion criteria were: 60 years or older, 2) ability to understand survey questions, and 3) informed consent.

All consenting participants were invited to a one-on-one, face-to-face interview in an office of the community health center after their health checkup. One community doctor conducted the interview after participating in a training workshop on the study design, interview protocol and recruitment and interview skills. After a brief introduction to the purpose of the study, participants were instructed to complete the following tasks: 1) describe their own health using the 3 L or 5 L questionnaire that was randomly assigned to them, 2) describe their own health using the SF-6D questionnaire, and 3) answer questions assessing socio-demographics and presence or absence of hypertension, hyperlipidemia, arthritis, osteoporosis, other chronic medical condition, and acute medical conditions. The average interview time per respondent was about 25 min.

### Instruments

The EQ-5D 3 L and 5 L self-reporting questionnaires describe health in five dimensions including mobility, self-care, usual activities, pain/discomfort, and anxiety/depression.

The 3 L version uses three response options (no problems, moderate problems, and extreme problems), and the 5 L version uses five response options (no problems, slight problems, moderate problems, severe problems, and extreme problems) [2]. The 3 L defines a total of 243 different health states, whereas the 5 L defines 3125 different health states. The Chinese 3 L and 5 L questionnaires have demonstrated good validity in multiple Chinese patient populations [7, 14]. The 3 L and 5 L utility scores were calculated using the 3 L and 5 L value sets for China [15, 16].

### Data analysis

The characteristics of participants who completed 3 L and 5 L questionnaires were compared using the chi-square test for categorical variables and the two-sample t test for continuous variables. The ceiling effects, descriptive efficiency, convergent validity, know-groups validity, and discriminatory power of the 3 L and 5 L measures were assessed separately.

Ceiling effects were assessed according to the proportion of participants reporting no problems in each of the EQ-5D dimensions. To facilitate interpretation, we estimated the absolute and relative reduction when going from 3 L to 5 L. The relative reduction (%) was computed by (Ceiling3 L-Ceiling5 L) / Ceiling3 L × 100.

Descriptive efficiency was assessed using the Shannon index (H′) and the evenness index (J’). H′ is defined as follows:

$$ {H}^{\prime }=-\sum \limits_{i=1}^L{\mathrm{p}}_i{\log}_2{\mathrm{p}}_i $$

Where H′ represents the absolute amount of informativity captured, L is the number of levels, and *p*_i_ is the proportion of observations in the i^th^ level (i = 1,...,L). In the case of an even (rectangular) distribution (i.e., all levels are equally filled), H′ reaches its maximum that equals log_2_L, which amounts to 1.58 to the 3 L (i.e., log_2_ 3) and 2.32 to the 5 L (i.e., log_2_ 5). J’ is a complementary index that reflects the extent to which the evenness distribution is achieved, and it is defined as: J’ = H′/H’_max_.

Convergent validity was tested by examining the correlation of the 3 L/5 L utility score with the SF-6D score using the Pearson’s correlation coefficient (r). The strength of the correlation was interpreted as strong (r > 0.50), moderate (0.35 ≤ r ≤ 0.50), weak (0.20 ≤ r < 0.35), or absent (r < 0.20). Known-groups construct validity was tested by comparing the 3 L/5 L utility scores of participants with and without metabolic syndrome (defined as having one or more of the five medical conditions: central obesity, high blood pressure, high blood sugar, high serum triglycerides, and low serum high-density lipoprotein), osteoporosis, arthritis, other chronic condition (defined as having heart disease, stroke, chronic kidney disease, chronic obstructive pulmonary disease, dementia, cancer, diabetes, stone, tracheitis, and/or cataract), and acute medical condition (defined as having cold, fever, diarrhea, fall, and/or injury). We hypothesized that participants with a chronic or acute condition would have lower utility scores than those without the condition [3].

Discriminatory power of the two EQ-5D utility scores was assessed in terms of their ability to distinguish participants with and without health conditions. The ratio of F statistics derived from the analysis of variance (ANOVA) tests of the two EQ-5D scores between participants with and without a condition was used. We computed the F statistic ratio in such a way that a ratio higher than 1.0 indicates that 5 L is more discriminative than 3 L.

Data were analyzed by using IBM SPSS Statistics for Windows, Version 22.0. Armonk, NY: IBM Corp (2013). All the statistics were two sided, and p<0.05 was considered statistically significant.

## Results

### Characteristics of participants

A total of 648 participants were enrolled. The mean (± standard deviation) age of the participants was 73.3 ± 6.4 years, with male being 55.7%. The majority of them were not formally educated (94.9%) and low level of income (68.4%). Table 1 shows the full characteristics of the entire sample and participants who completed the 3 L and 5 L questionnaires. There were no significant differences in demographic characteristics between two groups.

**Table 1:** Characteristics of participants

Characteristic	All participants (*n* = 648)	Participants who completed EQ-5D-3 L (*n* = 324)	Participants who completed EQ-5D-5 L (n = 324)	*P*-value
Age				0.244
Mean (SD)	73.25 (6.41)	72.95 (5.78)	73.54 (6.97)	
Median (IQR)	72.0 (8.00)	72.50 (6.00)	72.0 (9.00)	
Range	60.0 to 95.0	61.0 to 93.0	60.0 to 95.0	
Gender				0.937
Male	361 (55.7)	181 (55.9)	180 (55.6)	
Female	287 (44.3)	143 (44.1)	144 (44.4)	
BMI level				0.519
< 25	493 (76.1)	250 (77.2)	243 (75.0)	
≥ 25	155 (23.9)	74 (22.8)	81 (25.0)	
Education				0.372
Low (no/primary/secondary)	615 (94.9)	310 (95.7)	305 (94.1)	
High (tertiary/above)	33 (5.1)	14 (4.3)	19 (5.9)	
Marital status				0.279
Married	603 (93.1)	305 (94.1)	298 (92.0)	
Other (single/widowed/separated/divorced)	45 (6.9)	19 (5.9)	26 (8.0)	
Household income per month				0.151
< 2000 CNY	443 (68.4)	213 (65.7)	230 (71.0)	
≥ 2000 CNY	205 (31.6)	111 (34.3)	94 (29.0)	

Note EQ-5D EuroQol five-dimensional questionnaire, EQ-5D-3 L (3 L) three-level version of the EQ-5D, EQ-5D-5 L (5 L) five-level version of the EQ-5D

### Characteristics of responses to the EQ-5D questionnaires

For all of the dimensions, majority of the participants reported no problems (level 1) to both 3 L and 5 L (Table 2). For both 3 L and 5 L, the highest proportion of participants reported no anxiety/depression (3 L: 95.7%; 5 L: 84.3%), whereas the lowest proportion of participants reported no pain/discomfort (3 L: 56.2%; 5 L: 66.7%). Compared to 5 L, 3 L exhibited higher ceiling effects in the anxiety/depression dimension (absolute difference: 11.4%; relative difference: 11.9%). Ceiling effects between 3 L and 5 L were similar in the mobility, self-care and usual activities dimensions (range of absolute difference: − 3.8 to 4.0%; range of relative difference: − 5.2 to 5.0%), while lower ceiling effects of 3 L were found in the pain/discomfort dimension (absolute difference: − 10.5%; relative difference: − 18.7%). All dimensions considered, the proportion of participants reporting full health (11111) was 52.4 and 41.3% for 3 L and 5 L respectively, representing a reduction of 11.1 and 21.2% from 5 L to 3 L in absolute and relative term.

**Table 2:** Distributions of responses to EQ-5D-3 L and EQ-5D-5 L items

Dimension	EQ-5D-3 L		EQ-5D-5 L		Absolute difference in ceiling effects	Relative difference in ceiling effects
	Level	n (%)	Level	n (%)		
Mobility	1	240 (74.1)	1	238 (73.5)	0.6	0.8
	2	84 (25.9)	2	74 (22.8)		
	3	0.0	3	9 (2.8)		
			4	0 (0)		
			5	3 (0.9)		
Self-care	1	258 (79.6)	1	245 (75.6)	4.0	5.0
	2	65 (20.1)	2	72 (22.2)		
	3	1 (0.3)	3	7 (2.2)		
			4	0 (0)		
			5	0 (0)		
Usual activities	1	237 (73.1)	1	249 (76.9)	−3.8	−5.2
	2	87 (26.9)	2	67 (20.7)		
	3	0 (0.0)	3	6 (1.9)		
			4	0 (0)		
			5	2 (0.6)		
Pain/discomfort	1	182 (56.2)	1	216 (66.7)	−10.5	−18.7
	2	142 (43.8)	2	103 (31.8)		
	3	0 (0.0)	3	5 (1.5)		
			4	0 (0)		
			5	0 (0)		
Anxiety/depression	1	310 (95.7)	1	273 (84.3)	11.4	11.9
	2	14 (4.0)	2	50 (15.4)		
	3	0 (0.0)	3	1 (0.3)		
			4	0 (0)		
			5	0 (0)		
Full health (all dimensions considered)		170 (52.4)		134 (41.3)	11.1	21.2

Note EQ-5D EuroQol five-dimensional questionnaire

* Relative reduction was defined as ([Ceiling3 L-Ceiling5 L]/Celing3 L) in ceiling effects from the EQ-5D-3 L to the EQ-5D-5 L.

### Descriptive efficiency

The H′ values for 5 L were higher than those for 3 L for all dimensions as five levels led to a larger amount of information. The J’ values for 5 L were lower than those for 3 L for all but the anxiety/depression dimension (Table 3).

**Table 3:** Classification efficiency of the EQ-5D-3 L and the EQ-5D-5 L classification systems measured by Shannon index (H′)

Dimension	3 L		5 L	
	H′ (95% CI)	J’(95% CI)	H′(95% CI)	J’(95% CI)
Mobility	0.83	0.52	1.02	0.44
Self-care	0.75	0.48	0.91	0.39
Usual activities	0.84	0.53	0.91	0.39
Pain/discomfort	0.99	0.63	1.01	0.43
Anxiety/depression	0.26	0.16	0.65	0.28

Note CI confidence interval, EQ-5D EuroQol five-dimensional questionnaire, EQ-5D-3 L (3 L) three-level version of the EQ-5D, EQ-5D-5 L (5 L) five level version of the EQ-5D

* Larger H′ value indicates higher classification efficiency

**Table 4:** Known-groups validity and the discriminatory power of EQ-5D-3 L and EQ-5D-5 L

		EQ-5D-3 L		EQ-5D-5 L			F_5L_/F_3L_
	n (%)	Mean (SD)	F-statistic	n (%)	Mean (SD)	F-statistic	
Metabolic syndrome							
No	66 (20.4)	0.899 (0.144) *	2.742	67 (20.7)	0.952 (0.101) *	5.504	2.007
Yes	258 (79.6)	0.862 (0.169)		257 (79.3)	0.916 (0.113)		
Osteoporosis							
No	180 (55.6)	0.900 (0.152) *	14.635	181 (55.9)	0.955 (0.090) *	35.902	2.453
Yes	144 (44.4)	0.831 (0.172)		143 (44.1)	0.884 (0.124)		
Arthritis							
No	255 (78.7)	0.871 (0.174)	0.194	261 (80.5)	0.932 (0.115)	0.236	1.216
Yes	69 (21.3)	0.862 (0.126)		63 (19.5)	0.929 (0.090)		
Other chronic condition							
No	261 (80.6)	0.870 (0.161)	0.056	261 (80.5)	0.932 (0.108)	0.080	1.429
Yes	63 (19.4)	0.864 (0.179)		63 (19.5)	0.926 (0.125)		
Acute medical condition							
No	290 (89.5)	0.874 (0.163) *	2.411	306 (94.4)	0.926 (0.108) *	2.797	1.160
Yes	34 (10.5)	0.828 (0.176)		18 (5.6)	0.881 (0.158)		

Note EQ-5D EuroQol five-dimensional questionnaire, EQ-5D-3 L (3 L) three-level version of the EQ-5D, EQ-5D-5 L (5 L) five-level version of the EQ-5D, SD Standard deviation

* *p*<0.05(*t* tests of the difference between known groups)

### Construct validity

Pearson’s correlation coefficient was 0.608 (p<0.001) between 3 L and SF-6D and 0.433 (*p*<0.001) between 5 L and SF-6D. Generally speaking, participants without a medical condition had higher L/5 L score than those with the medical condition (Table 4). For example, The mean 3 L and 5 L scores for participants without osteoporosis (0.900 and 0.955) were higher than the mean 3 L and 5 L scores for those reporting to have osteoporosis (0.831 and 0.884) (*p*<0.001).

### Discriminatory power

The F-statistics derived from the known-groups comparisons were higher for 5 L than 3 L in each comparison (Table 4), suggesting that 5 L was more efficient or discriminative than 3 L. The F-statistic ratio ranged from 1.160 for comparison of participants with and without acute medical condition to 2.453 for comparison of participants with and without osteoporosis.

## Discussion

In this study, we compared the psychometric performance of the 3 L and 5 L EQ-5D instruments in a community-dwelling elderly Chinese population. Both instruments demonstrated validity. Furthermore, the 5 L may be preferable to the 3 L, as it exhibited lower ceiling effect and better discriminatory power. Our study design is unique in that we used an experiment design to compare the two EQ-5D instruments using two different groups. This is in contrast to the previous one-group design in which all subjects completed both 3 L and 5 L in a cross-sectional survey. Moreover, to the best of our knowledge, this is the first head-to-head comparison of preference-based HRQOL instruments in the fast growing elderly population. Thus, our study provides evidence for the appropriateness of switching from the 3 L EQ-5D to the 5 L EQ-5D for measuring the health outcomes of community-dwelling old people in China. The 3 L EQ-5D has been used in many population health surveys in the country [17–19]. Since the old population is rapidly increasing, the 5 L EQ-5D should be use in health surveys covering the entire adult Chinese population.

Consistent with previous studies [4, 20–23], on general populations, 5 L was found to have smaller ceiling effect as compared to 3 L. For general populations above 18 years old, magnitude of relative difference reported in this study was greater than those reported from Spain (1.62%) [20], England (15.3%) [21], USA (20.5%) [22] and South Korea (6.9%) [23] but less than that reported from Greece [4]. Being the only study conducted on middle-aged general population, the Greek study reported a lower percentage of respondents reporting full healthy on the 3 L(47.0%) and 5 L(31.2%) than our study did (52.4% for the 3 L and 41.3% for the 5 L), yielding a higher absolute (15.8% vs. 11.1%) and relative (33.7% vs. 21.2%) term respectively. Reported ceiling effect of present study being intermediate to previous studies could be due to factors such as true difference in the health status and cultural difference in answering health questions [24] between target populations.

As expected, extending the EQ-5D descriptive system from three to five levels led to a larger amount of information captured or higher absolute informativity. However, the relative informativity is slightly lower in some dimensions of 5 L, which have also been found in previous comparative studies [25–28]. The absolute and relative informativity of both EQ-5D versions in our study was lower than that in most of previous studies [4, 6, 29]. The relatively good health of our study sample, as evident by the very few endorsements of ‘severe’ or ‘extreme’ health problems with the EQ-5D questionnaires, could partly explain the relatively lower informativity observed in our study.

5 L demonstrated better discriminatory power than 3 L in this study, which is consistent with previous studies [3]. For example, a study of diabetic patients showed that 5 L differentiated between people with and without a complication or comorbid condition more efficiently than 3 L [7]. The better discriminatory power suggests that the 5 L is able to detect true between-group difference in health outcomes with a smaller sample size, compared with the 3 L. Therefore, the 5 L is preferred to the 3 L for measuring the health-related quality of life in the elderly Chinese population. It should be noted that the advantage of the 5 L over the 3 L as measured by the F-statistic ratio in our study is smaller than that in studies of patient populations. This could be because the participants were generally in good and they rarely endorsed the more severe levels of health problems, especially those from the 5 L. This limited the full capacity of the EQ-5D questionnaires especially for the 5 L because it contains more levels of severe health problems. The smaller advantage of the 5 L exhibited in our study could also be partly due to the fact that previous studies used a crosswalk algorithm to calculate the 5 L index score. Index scores based on crosswalk are less precise or reliable than scores based on value sets which are estimated using preferences directly elicited from representative general population samples [30].

Our study has some limitations. First, we did not assess important psychometric properties such as responsiveness and reliability, due to the cross-sectional design of the survey. The second limitation is that participants’ chronic conditions were self-reported without verification by clinicians. It is possible that some participants had undiagnosed medical conditions. Third, all participants in this study were recruited from one community of a small town in east China. Moreover, all participants were recruited from a health center. As a result, our study sample was fairly homogeneous and healthy. Therefore, it might not be representative and our findings may not be generalizable to the entire general elderly population in China. Last but not least, the between-subjects approach is more difficult to implement than the within-subjects approach because of sampling errors and the requirement of randomization. Future studies using this approach should take this limitation into account and perform a form sample size estimation.

## Conclusions

The study shows that both EQ-5D questionnaires are valid for measuring the health outcomes of the elderly in China. It appears that the EQ-5D-5 L is a more sensitive measure than the EQ-5D-3 L in this particular population. Therefore, the new EQ-5D questionnaire should be considered when there is a need to measure the health-related quality of life in the general elderly population in the country. Future research is needed to assess whether the EQ-5D-5 L is also more sensitive to change in this population.

## Data Availability

All data generated or analysed during this study are included in this published article.

## Abbreviations

**HRQOL**: health related quality of life
**EQ-5D**: EuroQol five-dimensional questionnaire
**EQ-5D-3 L (3 L)**: three-level version of the EQ-5D
**EQ-5D-5 L (5 L)**: five-level version of the EQ-5D
**CI**: confidence interval
**SD**: Standard deviation
